# Size of cup affects the anterior capsular distance in total hip arthroplasty, as measured with ultrasound

**DOI:** 10.1186/1471-2474-15-23

**Published:** 2014-01-20

**Authors:** Sarunas Tarasevicius, Valdemaras Loiba, Justinas Stucinskas, Otto Robertsson, Hans Wingstrand

**Affiliations:** 1 Department of Orthopedics, Lund University Hospital, Getingevagen 4, Lund 22185, Sweden; 2 Departments of Orthopedics, Lithuanian University of Health Sciences, Eiveniu 2, Kaunas LT-50009, Lithuania

**Keywords:** Sonography, Cup size, Capsular distance, THA

## Abstract

**Background:**

Previously was found that sonography is a reliable method to measure a capsular distance in total hip arthroplasty hips. The aim of our current study was to investigate the relation between the implanted size of the cup and the anterior capsular distance, as measured with ultrasound one year after THA.

**Methods:**

50 osteoarthritis (OA) patients operated on with total hip arthroplasty one year before were included in the study and the anterior capsular distance was measured sonographically. Patients were grouped with respect to cup size. The correlation between the implanted cup size and capsular distance was determined.

**Results:**

The mean capsular distance in the whole group was 1.37 (SD 0.19) cm. The mean capsular distance in the group with small cups was 1.27 (SD 0.13) cm, in large cups it was 1.45 (SD 0.20) cm, p = 0.02. Spearman correlation analysis showed a statistically significant correlation between a greater capsular distance and the larger size of the cup (r = 0.5, p < 0.0001).

**Conclusion:**

The greater capsular distance in successful THA hips is affected by cup size. We propose that this should be considered when evaluating sonography of the anterior capsular distance after THA.

## Background

Ultrasonography (US) is a valuable tool for diagnosing capsular distension in the hip joint and has been used for diagnosing synovitis, infection and hemarthrosis [1-3]. It has also been used in research, e.g. to demonstrate a correlation between radiographic signs of wear and loosening and increased capsular distance in cemented total hip arthroplasty (THA) [4-6]. Thus, measuring the capsular distance after THA may provide useful clinical information necessary for diagnostic assessment.

Previously we investigated the reliability of US in vitro to measure the capsular distance in a plastic model. Subsequently, we used US measurements from 22 THA patients, and by 3 different examiners, to calculate intra- and interobserver agreements [7]. We concluded that ultrasonography is a reliable method for measuring anterior capsular distance in THA, especially if performed by an experienced examiner. However, there is a lack of information whether the US measurements may be affected by factors such as the size of the implanted components.

The aim of our study was to analyze the relation between the implanted size of the cup and the anterior capsular distance, as measured with US in patients one year after successful THA.

## Methods

We examined 50 primary non inflammatory osteoarthritis (OA) patients operated on with THA during the course of 6 months that one year after surgery had no or minimal pain, and that were not using pain relieving drugs. All the patients had received the same type of standard offset stem (DePuy, C-stem) with a 28 mm femoral head together with an all-poly cemented cup (DePuy, Ultima). All the patients had been operated on using posterior approach leaving the anterior capsule intact. The patients were divided into two groups; Group A consisting of those with 44, 46 and 48 mm outer diameter cups and group B with 54, 56 and 58 mm cups.

**Figure 1:**
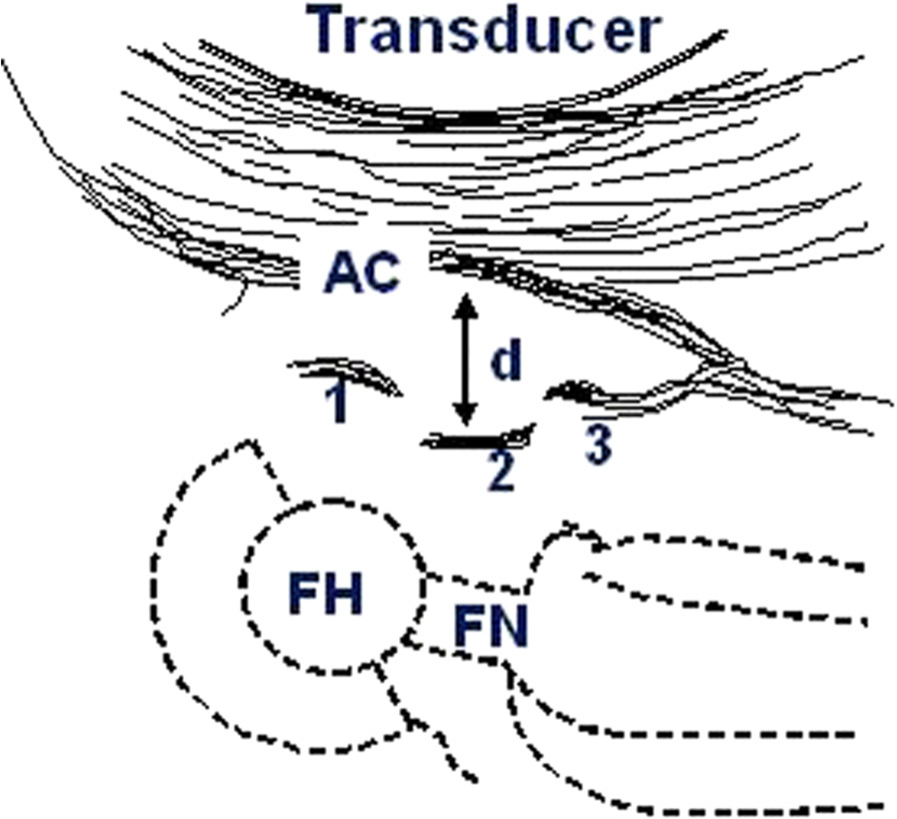
**Schematic drawing showing sonographic findings in THA patients.** FH: prosthetic femoral head; FN: prosthetic femoral neck; AC: anterior capsule; d is the distance between the anterior capsule and the prosthetic femoral neck; 1 is the echo from the prosthetic femoral head (FH); 2 is the echo from the prosthetic femoral head (FN); 3 is the echo from the prosthetic collar (not always present).

**Figure 2:**
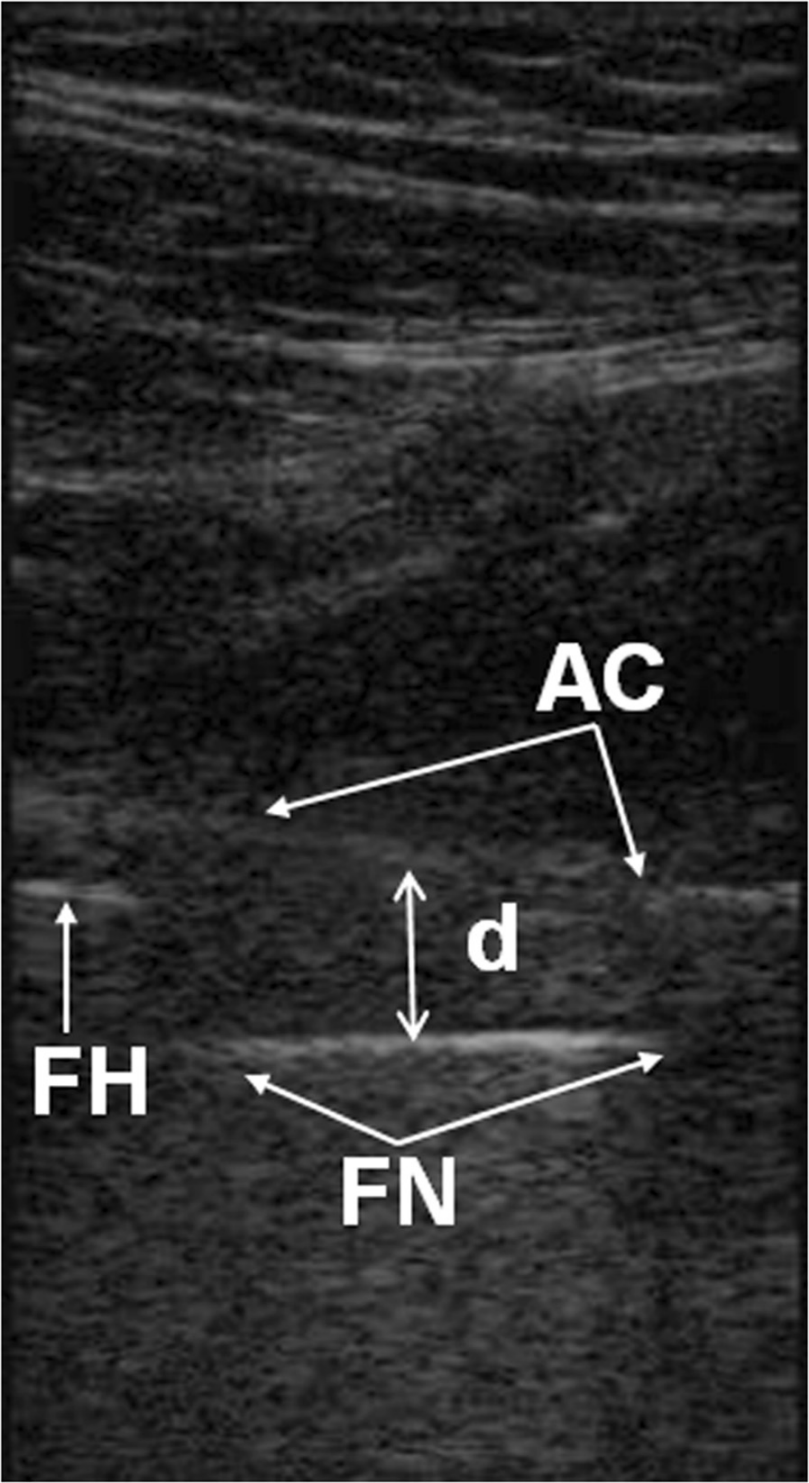
**Sonographic measurement of the capsular distance in THA.** AC - echo from the anterior surface of the anterior hip joint capsule. FN - echo from the anterior surface of the prosthetic femoral neck. FH – echo from the anterior surface of the prosthetic femoral head. d - represents the distance between anterior capsule and prosthetic femoral neck.

12–13 months after their surgery a US examination was performed. A portable Sonosite 180 L38 (Sonosite) apparatus with 5–10 Mhz linear transducer was used for all examinations. All the measurements were performed by one researcher with the patient supine in a sagittal plane, from the anterior aspect of the hip along the axis of the femoral neck. The anterior capsular distance was defined as the distance between the metallic echo from the mid part of the anterior surface of the prosthetic femoral neck perpendicular to the echo from the inner layer of the anterior capsule (Figures 1 and 2).

The primary effect variable, used for power calculation analysis, was the capsular distance. With an assumption of a difference in means of 3 mm, and an SD (standard deviation) of 3 mm for both groups, and aiming at a power of 0.80 and a risk of 0.05 for type-1 error, 16 patients were required in each group. Age, capsular distance, were compared between groups using the Mann–Whitney U test The Spearman correlation (r) was used to calculate the correlation between variables. A p-value of < 0.05 was considered significant. SPSS software was used for the calculations.

The study was approved by the ethical committee of the Lithuanian University of Health Sciences hospital, Kaunas Clinics (BE-2-19). Informed consent was obtained from each subject in writing before study and only patients older than 18 years were included.

## Results and discussion

Group A with the smaller cups included 22 patients of which 2 were males, while group B included 28 patients of which 23 were males (Table 1).

**Table 1:** Patients related data, in small and large THA cup groups (n = 50)

**Variables**	**Group A, n = 22**	**Group B, n = 28**	**p-value**
Age	73 (SD 8)	66 (SD 12)	0.02
Gender	M-2, F-20	M-23, F-5	<0.001

The mean capsular distance for the whole group was 1.37 (SD 0.19) cm. The mean capsular distance in group A was 1.27 (SD 0.13) cm and varied from 1.02 – 1.45 cm, in group B it was 1.45 (SD 0.20) cm and varied from 1.16 – 2.16 cm, p = 0.02. Mean capsular distance for males was 1.46 (SD 0.20) cm, for females 1.28 (SD 0.14) cm, p = 0.001. Spearman correlation analysis showed a statistically significant correlation between the greater capsular distance and the larger size of the cup (r = 0.5, p < 0.0001). No significant correlation was observed between stem size and capsular distance (p = 0.3).

In a healthy hip there is no measurable distance between the femoral neck and the inner layer of the anterior capsule as the amount of joint fluid is minimal [8]. However, due to the relatively narrow femoral neck in THA there will be room between the neck and an intact anterior capsule immediately after surgery which does not fill up completely with scar tissue, at least not in during the course of one year, as shown by this and previous studies on well-functioning hips [6].

We established that the anterior capsular distance as measured with US at 1 year after the surgery is affected by the size of the cup, but not by the size of the femoral stem.

Our findings are explained by the geometry of the hip since the proximal part of the capsule is attached to the borders and the roof of the acetabulum, while the distal part of the capsule is attached to the intertrochanteric region of the femur. An increase in the acetabular cup size increases the radius of the capsular attachment which in turn increases the distance between the metal femoral neck and the capsule in all directions. Also it was reported that greater sonographically measured “capsular distance” is correlated to increased linear and volumetric wear in radiographically stable THA hips [6] and it is in concordance with other literature report that acetabular cup size may lead to the higher risk of the implant failure [9].

We wound a mean capsular distance of 1.37 cm for the whole group, which is in concordance with our previous findings where we similarly investigated THA patients one year postoperatively and found mean capsular distance of 1.34.

Thus, according our studies we define the normal values of capsular distance but taking into account the cup size.

## Conclusion

We conclude that the greater capsular distance in successful THA hips is affected by cup size and propose that this should be considered when evaluating sonography of the the anterior capsular distance after THA.

## Abbreviations

OA: Osteoarthritis; US: Ultrasonography; THA: Total hip arthroplasty; SD: Standard deviation.

## Competing interests

The authors declare that they have no competing interests.

## Authors’ contributions

ST: data collection, compilation and analysis, writing manuscript, VL: data collection, editing manuscript, JS: data collection, editing manuscript, OR: statistical analysis, editing manuscript, HW: organizing study, editing manuscript. All authors read and approved the final manuscript.

## Pre-publication history

The pre-publication history for this paper can be accessed here:

http://www.biomedcentral.com/1471-2474/15/23/prepub
